# How to Handle Concomitant Asymptomatic Prosthetic Joints During an Episode of Hematogenous Periprosthetic Joint Infection, a Multicenter Analysis

**DOI:** 10.1093/cid/ciaa1222

**Published:** 2020-08-18

**Authors:** Marjan Wouthuyzen-Bakker, Marine Sebillotte, Cédric Arvieux, Marta Fernandez-Sampedro, Eric Senneville, José Maria Barbero, Jaime Lora-Tamayo, Craig Aboltins, Rihard Trebse, Mauro José Salles, Tobias Siegfried Kramer, Matteo Ferrari, Joaquín Garcia-Cañete, Natividad Benito, Vicens Diaz-Brito, Maria Dolores del Toro, Matthew Scarborough, Alex Soriano

**Affiliations:** Department of Medical Microbiology and Infection Prevention, University of Groningen, University Medical Center Groningen, the Netherlands; Department of Infectious Diseases and Intensive Care Medicine, Rennes University Hospital, Rennes, France; Department of Infectious Diseases and Intensive Care Medicine, Rennes University Hospital, Rennes, France; Great West Reference centers for Complex Bone and Joint Infections (CRIOGO), Rennes, France; Service of Infectious Diseases, Hospital Universitario Marqués de Valdecilla, University of Cantabria, Instituto de investigación sanitaria Valdecilla (IDIVAL), Santander, Spain; Department of Infectious Diseases, Lille, University Hospital Gustave Dron Hospital, Tourcoing, France; Department of Internal Medicine. Hospital Universitario Principe de Asturias, Madrid, Spain; Department of Internal Medicine. Hospital Universitario 12 de Octubre. Instituto de Investigación i+12. Madrid, Spain; The Department of Infectious Diseases, Northern Health, Melbourne, Australia; The University of Melbourne, Northern Clinical School, Melbourne, Australia; Service for Bone Infections, Valdoltra Orthopaedic Hospital, the Faculty of Medicine, University of Ljublijana, Ankaran, Slovenia; Santa Casa de São Paulo School of Medical Sciences and Musculoskeletal infection group, Federal University of São Paulo, Brasil; Institute for hygiene and environmental medicine Charité-Universitätsmedizin Berlin, Germany; Evangelisches Waldkrankenhaus Spandau, BerlinGermany; Department of Biomedical Sciences, Humanitas University, Milan, Italy; Istituto di Ricovero e Cura a Carattere Scientifico (IRCCS) Humanitas Research Hospital, Milan, Italy; Department of Internal Medicine-Emergency, Instituto de Investigación Sanitaria (IIS)-Fundación Jiménez Díaz, Universidad Autónoma de Madrid (UAM), Madrid, Spain; Infectious Diseases Unit, Hospital de la Santa Creu i Sant Pau, Institut d’Investigació Biomèdica Sant Pau, Barcelona, Spain; Department of Medicine. Universitat Autònoma de Barcelona, Spain; Infectious Diseases Unit, Parc Sanitari Sant Joan de Deu, Sant Boi, Barcelona, Spain; Unidad Clínica de Enfermedades Infecciosa y Microbiología. Universidad de Sevilla. Instituto de Biomedicina de Sevilla (IBIS), Sevilla, Spain; Bone Infection Unit, Nuffield Orthopaedic Centre, Oxford University Hospitals National Health Service (NHS) Foundation Trust, Oxford, England; Service of Infectious Diseases, Hospital Clínic, University of Barcelona, Barcelona, Spain

**Keywords:** periprosthetic joint infection, late acute, hematogenous, asymptomatic, bacteremia

## Abstract

**Background:**

Prosthetic joints are at risk of becoming infected during an episode of bacteremia, especially during *Staphylocococcus aureus* bacteremia. However, it is unclear how often asymptomatic periprosthetic joint infection (PJI) occurs, and whether additional diagnostics should be considered.

**Methods:**

In this multicenter study, we retrospectively analyzed a cohort of patients with a late acute (hematogenous) PJI between 2005–2015 who had concomitant prosthetic joints in situ. Patients without at least 1 year of follow-up were excluded.

**Results:**

We included 91 patients with a hematogenous PJI and 108 concomitant prosthetic joints. The incident PJI was most frequently caused by *Staphylococcus aureus* (43%), followed by streptococci (26%) and Gram-negative rods (18%). Of 108 concomitant prosthetic joints, 13 were symptomatic, of which 10 were subsequently diagnosed as a second PJI. Of the 95 asymptomatic prosthetic joints, 1 PJI developed during the follow-up period and was classified as a “missed” PJI at the time of bacteremia with *S. aureus* (1.1%). Infected prosthetic joints were younger than the noninfected ones in 67% of cases, and prosthetic knees were affected more often than prosthetic hips (78%).

**Conclusions:**

During an episode of hematogenous PJI, concomitant asymptomatic prosthetic joints have a very low risk of being infected, and additional diagnostic work-up for these joints is not necessary.

## Teaser

We evaluated a cohort of patients with hematogenous periprosthetic joint infection and concomitant asymptomatic prosthetic joints. We demonstrate that if a prosthetic joint is asymptomatic, the chance of infection is very low. These joints do not require additional diagnostic work-up.

A serious complication of total joint replacement is the development of a periprosthetic joint infection (PJI). This complication occurs in around 1–2% of patients after primary arthroplasty and around 10% after revision arthroplasty [1–3]. Infection mostly occurs in the postsurgical period, but may develop at a later stage as well, either as a late chronic or late acute PJI [4, 5]. According to registry observations, patients with a prosthetic joint in situ are at risk for developing a late acute (hematogenous) PJI at a rate of approximately 0.07% per life year of the prosthesis, with the highest risk observed in knees [6]. Moreover, during an episode of bacteremia, especially in the case of *Staphylocococcus aureus* bacteremia, it has been suggested that prosthetic joints have a 20–30% chance of becoming secondarily infected [7–12]. It is well established that an additional diagnostic work-up and early surgical debridement are required in symptomatic joints with a high clinical suspicion of PJI, but it is unclear how an asymptomatic joint should be approached in this regard. A missed diagnosis of an acute PJI may result in the development of a chronic PJI, treatment of which is likely to mandate removal of the implant [13]. Timely diagnosis is thus of extreme importance. Patients who are diagnosed with a PJI and who have concomitant prosthetic joints in situ appear to have the highest risk for having a second PJI [14–16]. Therefore, we retrospectively analyzed a cohort of patients with hematogenous PJI who also had 1 or more concomitant prosthetic joints in situ at the time of PJI diagnosis. We evaluated how many asymptomatic joints were diagnosed with PJI at the time of presentation and how many developed a PJI during the follow-up period.

## MATERIAL AND METHODS

### Study Design and Inclusion Criteria

This study was performed as part of a large, international, multicenter observational study in which data from consecutive patients with hematogenous PJI between January 2005 and December 2015 were collected and retrospectively evaluated. A PJI was defined according to the adapted diagnostic criteria of the Musculoskeletal Infection Society [17], requiring at least 2 positive, intraoperative tissue cultures with phenotypically identical microorganisms. A hematogenous PJI was defined by the sudden onset of symptoms and by signs of arthritis occurring more than 3 months after the index arthroplasty in a previously asymptomatic prosthetic joint. Patients with a sinus tract, culture-negative PJIs, and a follow-up of less than 1 year were excluded. Patients were included in the final analysis if they had at least 1 other concomitant prosthetic joint in situ at the time of clinical presentation. Informed consent was obtained when required by the ethics committee of the participating center. Variables relating to patient characteristics, clinical presentation, diagnostics, microbiology, surgical intervention, antibiotic treatment, and outcome were collected and analyzed.

### Statistical Analysis

A chi-square test (or a Fisher exact test, when appropriate) was used to analyze the difference between groups for categorical variables, and a student *t*-test (or Mann-Witney U test, when data were not normally distributed) was used for continuous variables. All analyses were 2-tailed and *P* values < .05 were considered statistically significant. The statistical analysis was performed using SPSS, version 23.0 (SPSS Inc., Chicago, IL).

## RESULTS

**Figure 1.**
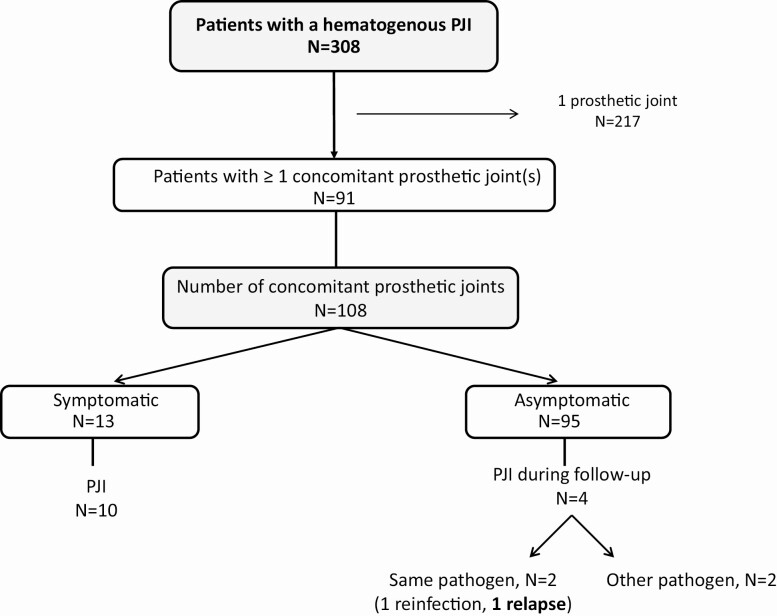
Flow chart for inclusion. Abbreviation: PJI, periprosthetic joint infection.

Figure 1 shows the inclusion flowchart of patients with a hematogenous PJI and the number of concomitant prosthetic joints. Of 308 consecutive patients with hematogenous PJIs, 91 (29.5%) had at least 1 additional prosthetic joint in situ, resulting in a total of 108 concomitant prostheses for inclusion in the final analysis (1 joint each in 77 patients, 2 joints each in 11 patients, and 3 joints each in 3 patients).

### Characteristics of Hematogenous PJIs

Table 1 shows the baseline and clinical characteristics of the 91 patients. The hematogenous PJI episode mainly manifested in knees (69%), and the infection was most commonly due to *Staphylococcus aureus* (43%), followed by streptococci (26%) and Gram-negative rods (18%). The portal of entry for the bacteremia was identified in 46% of cases, and endocarditis was diagnosed in 5%. Patients with *S. aureus* PJI received an echocardiogram in 17/39 (44%) cases. At the time of clinical presentation, blood cultures were positive in 32/91 cases (35%) and negative in 29/91 cases (32%), and no blood cultures were obtained in 30/91 cases (33%). Most of the patients (77%) were treated with debridement, antibiotics, and implant retention.

**Table 1.** Patient Characteristics in Hematogenous Periprosthetic Joint Infection

Patient characteristics, n = 91	n*/*N, %
Baseline characteristics	
Gender, male	37/91, 40.7%
Age >80 years	23/91, 25.3%
BMI >30	29/56, 51.8%
ASA classification ≥III	35/78, 44.9%
Medical history	
Hypertension	48/90, 53.3%
Diabetes mellitus	16/88, 18.2%
Ischemic heart disease	14/91, 15.4%
Rheumatoid arthritis	13/91, 14.3%
Heart failure	9/91, 9.9%
COPD	8/91, 8.8%
Chronic renal insufficiency	7/91, 7.7%
Active malignancy	6/91, 6.6%
Liver cirrhosis	3/91, 3.3%
Medication	
Oral anticoagulant	16/91, 17.6%
Immune-suppressive drugs	14/91, 15.4%
Characteristics infected prosthetic joint	
Knee	63/91, 69.2%
Hip	26/91, 28.6%
Indication prosthesis:	
Osteoarthritis	71/91, 78%
Rheumatoid arthritis	12/91, 13.2%
Fracture	4/91, 4.4%
Revision prosthesis	30/90, 33.3%
Cemented stem	45/60, 75%
Clinical presentation	
Duration of symptoms >10 days	33/85, 38.8%
Temperature >38.5°C	20/85, 23.5%
CRP >150 mg/L	45/79, 56.9%
Leucocytes >17 cells/ µL	11/85, 12.9%
Positive blood cultures	32/91, 35.1%
Negative blood cultures	30/91, 32.9%
No blood cultures taken	29/91, 31.8%
Endocarditis	3/91, 3.3%
Portal of entry bacteremia identified	42/91, 46.1%
Microorganism	
*Staphylococcus aureus*	38/91, 41.8%
Methicillin resistant	5/91, 5.5%
*Streptococcus* species	23/91, 25.3%
Gram-negative rods	14/91, 15.4%
*Enterococcus* species	5/91, 5.5%
Surgical strategy	
DAIR	70/91, 76.9%
More than 1 DAIR performed	13/70, 18.6%
Mobile components exchanged	30/70, 42.8%
Revision surgery	18/91, 19.8%
No surgery	3/91, 3.3%

Abbreviations: ASA, American Society of Anesthesiologist; BMI, body mass index; COPD, chronic obstructive pulmonary disease; CRP, C-reactive protein; DAIR, debridement, antibiotics, and implant retention.

### Analysis of Concomitant Prosthetic Joints

Of the 91 patients with a hematogenous PJI, 108 concomitant prosthetic joints were analyzed, including 56 knees, 44 hips, 3 shoulders, and 5 other joints. Of these, 13/108 (12%) were symptomatic at the time of presentation and 95/108 (88%) were asymptomatic (Figure 1). Of the 13 symptomatic joints, 7 patients experienced acute pain of the joint (54%), 2 patients experienced acute or chronic pain (15%), and 4 patients experienced chronic pain (31%). All of the 13 symptomatic joints had clinical signs of infection on physical examination, and 10 of these 13 patients were diagnosed as having a second PJI (77%). In the other 3 patients, a PJI was ruled out: all of these had chronic pain, which was attributed to mechanical reasons.

The median time of follow-up of the 95 asymptomatic joints was 52 months (range, 13–130 months). There were 4 cases that developed a PJI during follow-up (4.2%); all were caused by *S. aureus*. In 2 out of 4 cases, the original PJI was caused by *Streptococcus pyogenes*, and was thus considered as a new hematogenous infection. In the other 2 cases, *S. aureus* was the causative microorganism in the original PJI as well. These 2 cases were evaluated in more detail to decipher whether these were potential, unrecognized infections at the time of the first PJI episode. The first patient, with a bilateral knee prosthesis, presented in February 2008 with a hematogenous PJI of the right knee caused by *S. aureus*. Blood cultures were positive during clinical presentation. After a failed surgical debridement, the patient underwent a 2-stage exchange procedure and was treated with antibiotics accordingly. The patient presented with an acute PJI of the left knee prosthesis 4 years later, also due to *S. aureus*. Although the antibiogram was the same, as the patient had not experienced any chronic pain during this 4-year interval, the infection was considered as a new hematogenous PJI and not as a relapse of an unrecognized infection in 2008. The second patient was diagnosed in September 2011 with a late acute *S. aureus* PJI of the right hip. This patient had positive blood cultures at presentation. Endocarditis was excluded by transesophageal echocardiography. The patient was successfully managed with surgical debridement and a rifampin-based antibiotic regimen, consisting of an intravenous beta-lactam antibiotic, during the first 4 weeks, and was treated after that with fusidic acid as the final codrug of rifampin. The left hip remained asymptomatic at that time. However, after stopping antibiotic treatment in February 2012, the left hip became symptomatic and *S. aureus* was subsequently cultured in this joint, with an identical antibiogram as the initial infection (both a penicillin-sensitive strain). This PJI was considered as a relapse of a previously undiagnosed infection.

Altogether, only 1 out of the 95 asymptomatic prosthetic joints was classified as an unrecognized PJI at the time of the first clinical presentation (1.1%). When considering only *S. aureus* PJIs, this percentage was 2.7% (1 out of 37); in those with documented *S. aureus* bacteremia, it was 5.6% (1 out of 18).

Of the 91 patients with at least 1 asymptomatic joint in situ, 11 received additional diagnostics: 1 patient underwent joint aspiration and 10 patients underwent nuclear imaging (fluuorodeoxyglucose [FDG]-positron emission tomography [PET] computed tomography in 4 cases and white blood cell scintigraphy in 6 cases). In all 11 cases, there were no signs of PJI on imaging and synovial fluid analysis, and none of these patients were diagnosed with a PJI during follow-up.

### Which Prosthetic Joint Gets Infected

**Figure 2.**
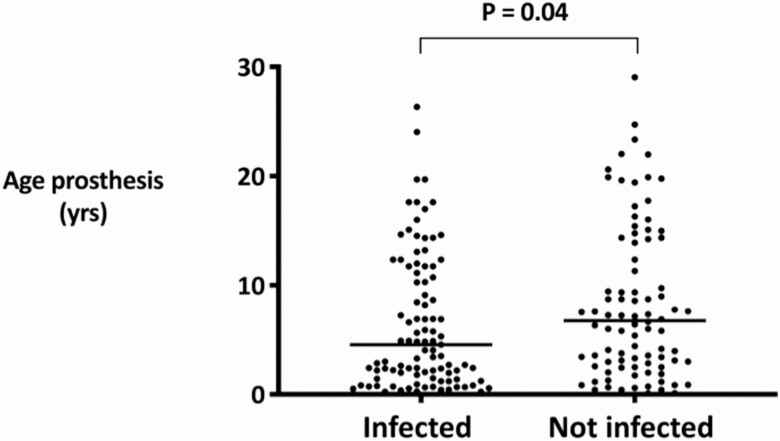
Prosthesis age and occurrence of hematogenous PJI. Abbreviation: PJI, periprosthetic joint infection.

We additionally investigated whether the type of joint or the age of the prosthesis influenced the risk of infection. In the majority of cases (67%), the infected prosthetic joint was younger than the noninfected joint, with a median prosthesis age of 4.5 (interquartile range, 1.5–11.8) versus 6.7 (interquartile range, 2.8–14.3) years, respectively (*P* = .04; Figure 2). In 27 patients with both knee and hip prostheses in situ (*n* = 27), the knee prosthesis became infected in 21/27 (78%) cases, and the hip prosthesis became infected in 6/27 (22%). In no cases were both joints infected.

## Discussion

The management of concomitant asymptomatic implants in patients with a documented PJI is not widely addressed in literature. Timely diagnosis of PJI is important, in particular for hematogenous PJIs, where early surgical debridement without removing the implant is commonly recommended. In our analysis of 91 patients with a hematogenous PJI and 108 concomitant prosthetic implants, we demonstrate a very low incidence (1.1%) of a second PJI in prosthetic joints that have no clinical signs or symptoms of infection. Although we cannot rule out the possibility that asymptomatic infection is being eradicated by the antibiotic treatment used for the index PJI, our results suggest that performing additional diagnostics or surgical interventions is not indicated in asymptomatic prosthetic joints.

Our results are supported by previous analyses. Tande et al [7] evaluated 85 patients with a *S. aureus* bacteremia who had a prosthetic joint in situ. A PJI was diagnosed in 39/139 (28%) arthroplasties, and 38 of these had clinical signs of infection. In line with our results, of the 100 cases in which a PJI was not diagnosed, only 4% of patients developed a PJI during follow-up, and all were caused by *S. aureus*. Since the range of developing a PJI during follow-up varied between 174 and 670 days, it is unclear whether these PJIs represented a relapse of infection or a new hematogenous infection with the same microorganism, which would be reasonable in persistent nasal carriers of *S. aureus* [18, 19]. Dufour et al [8] recently described a similar observation. Out of the 143 evaluated arthroplasties in their study, 19% developed a PJI during an episode of *S. aureus* bacteremia. All of these patients experienced pain or swelling of the affected joint during their hospital stay. No additional PJIs occurred after a median follow-up of 261 days in the asymptomatic prosthetic joints. As with our data, these articles suggest that although the risk of developing a PJI during *S. aureus* bacteremia is high, it is unlikely that asymptomatic implants are infected and that diagnostic measures on these joints can reasonably be avoided.

The observation that knees are more likely to become infected than other joints is replicated in several observational studies on hematogenous PJIs [6, 7, 20]. The finding of younger implants being more prone to infection was also reported by Honkanen et al [12], demonstrating that the risk for developing a PJI was highest for bacteremias occurring within a year of surgery. However, it should be noted that in this particular study, PJIs occurring within the first 3 months after the index surgery were included, making it difficult to determine whether the PJI was the cause or the consequence of the bacteremia.

Our study has a few limitations. First, we analyzed only patients with a hematogenous PJI who had at least 1 other prosthetic joint in situ. However, it is known that patients with a history of PJI have a higher chance of having a second PJI, especially during an episode of bacteremia [14–16]. Thus, even in this “high-risk” group, the chance of having a PJI in an asymptomatic joint at the time of having a hematogenous PJI is very low. A further limitation of our study is that not all hematogenous PJIs had positive blood cultures at the time of clinical presentation. Although this may call into question the hematogenous origin of infection, all diagnosed cases had a sudden onset of acute symptoms and signs of arthritis, and were entirely asymptomatic prior to clinical presentation in the hospital. This, together with the microorganisms isolated (which were mainly virulent ones: *S. aureus*, ß-hemolytic streptococci, and Gram-negative rods), makes a hematogenous origin highly likely.

In conclusion, in patients with bacteremia or with a hematogenous PJI, all concomitant prosthetic joints should be carefully examined. If symptoms or signs of infection are present, appropriate additional diagnostics should be undertaken. If clinical symptoms of infection are absent, the chance of PJI is very low and additional diagnostics are unnecessary.

## Note

***Potential conflicts of interest.*** T. S. K. reports personal fees from Infectopharm, outside the submitted work. R. T. has received consulting fees from Medacta, speaker fees from Zimmer Biomet and Link, and grants from Ceramtec, and is President of European Bone and Joint Infection Society, outside the submitted work. All other authors report no potential conflicts. All authors have submitted the ICMJE Form for Disclosure of Potential Conflicts of Interest. Conflicts that the editors consider relevant to the content of the manuscript have been disclosed.
